# Past-directed scalar field gradients and scalar-tensor thermodynamics

**DOI:** 10.1007/s10714-023-03095-7

**Published:** 2023-03-09

**Authors:** Andrea Giusti, Serena Giardino, Valerio Faraoni

**Affiliations:** 1 Institute for Theoretical Physics, ETH Zurich, Wolfgang-Pauli-Strasse 27, 8093 Zurich, Switzerland; 2 Max Planck Institute for Gravitational Physics (Albert Einstein Institute), Callinstraße 38, 30167 Hannover, Germany; 3 Institute for Theoretical Physics, Heidelberg University, Philosophenweg 16, 69120 Heidelberg, Germany; 4 Department of Physics and Astronomy, Bishop’s University, 2600 College Street, Sherbrooke, QC J1M 1Z7 Canada

**Keywords:** Modified gravity, Scalar-tensor gravity, Thermodynamics, Scalar field

## Abstract

We refine and slightly enlarge the recently proposed first-order thermodynamics of scalar-tensor gravity to include gravitational scalar fields with timelike and past-directed gradients. The implications and subtleties arising in this situation are discussed and an exact cosmological solution of scalar-tensor theory in first-order thermodynamics is revisited in light of these results.

## Introduction

There are several motivations to extend the theory of gravity beyond Einstein’s General Relativity (GR). All attempts to reconcile this theory with quantum physics introduce deviations from GR in the form of extra fields, higher-order equations of motion, or higher-order curvature invariants. For example, taking the low-energy limit of the bosonic string theory, the simplest among string theories, produces $\omega =-1$ Brans-Dicke theory instead of GR, which is the prototype of a scalar-tensor theory (and $\omega$ is the Brans-Dicke coupling) [1, 2].

However, the most compelling motivations to study alternative theories of gravity come from cosmology. For instance, the inflationary model most favoured by data, namely Starobinsky inflation, includes quantum corrections to GR. Most importantly, a satisfactory understanding of the present-day accelerated expansion of the universe is lacking within the realm of the standard $\Lambda$CDM model of cosmology based on GR: it requires one to introduce an astonishingly fine-tuned cosmological constant or another form of *ad hoc* dark energy, whose nature remains elusive [3].

In any case, even admitting the presence of dark energy still leaves other problems of $\Lambda$CDM unresolved, such as the Hubble tension [4, 5], the requirement for an equally mysterious dark matter, and the singularity problem that plagues cosmology and black hole physics. It is at least reasonable, therefore, to study alternative theories of gravity to resolve or alleviate these issues.

The simplest way to modify GR consists of adding a scalar (massive) degree of freedom, which resulted in Brans-Dicke gravity [6] and its scalar-tensor generalizations [7–10]. The class of *f*(*R*) theories of gravity, which turns out to be a subclass of scalar-tensor theories, is extremely popular to explain the present cosmic acceleration without dark energy ( [11], see [12–14] for reviews). In the last decade, the old Horndeski gravity [15] has been revisited and studied intensively (see [16] for a review). This class of theories was believed to be the most general scalar-tensor gravity admitting second-order equations of motion, but then it was discovered that, if a suitable degeneracy condition is satisfied, the even more general Degenerate Higher Order Scalar-Tensor (DHOST) theories admit second-order equations of motion (see [17] for a review).

Horndeski and DHOST theories contain arbitrary functions in their actions that make the field equations very cumbersome and their study difficult. The multi-messenger event GW170817/GRB170817, [18, 19] confirming that gravitational wave modes propagate at the speed of light, has essentially ruled out Horndeski theories with the most complicated structure [20], but many possibilities (corresponding to four free functions in the action) remain. As a result, it is difficult to grasp the detailed physical meaning of these theories and their solutions and much of the work necessarily remains confined to formal theoretical aspects and to the search for analytical solutions.

In an attempt to gain physical intuition for scalar-tensor gravity (including viable Horndeski theories), it is fruitful to interpret it through an effective fluid description in which the (Jordan frame) field equations are written as effective Einstein equations and the remaining geometrical terms, when moved to the right-hand side, form the stress-energy tensor of an effective *dissipative* fluid [21–24]. This effective fluid description is possible when the gradient of the scalar field degree of freedom $\phi$ of the theory is timelike [21–23].

In this context, using the three constitutive relations postulated in Eckart’s first-order thermodynamics of dissipative fluids [25], we were able to introduce an effective “temperature of gravity” and shear and bulk viscosity coefficients [24, 26, 27]. Armed with these concepts, one can describe GR as the state of thermal equilibrium of gravity corresponding to zero temperature and scalar-tensor gravity as a state of non-equilibrium at higher temperature (this temperature being relative to GR) [24, 26, 27].^1^ Dissipation corresponds to the relaxation of this effective fluid toward the GR state of equilibrium.

We applied the recent first-order thermodynamics of modified gravity to Friedmann-Lemaître-Robertson-Walker (FLRW) cosmology [30] and additionally searched for possible alternative equilibrium states, which turned out to be metastable [31, 32]. We also found that the alternative description of scalar-tensor gravity in the Einstein conformal frame swaps temperature with chemical potential [33].

These early studies do not adequately discuss a potential limitation of the formalism, *i.e.*, the fact that the scalar field gradient must be future-directed for it to be meaningful.

Here we refine the previous discussions [27, 30] to make this limitation explicit and we discuss a possible extension of the formalism to situations in which this gradient is timelike but past-directed, which does indeed occur in certain analytical solutions of scalar-tensor theories. We follow the notations of Ref. [34]: the metric signature is ${-}{+}{+}{+}$ and we use units in which the speed of light *c* and Newton’s constant *G* are unity.

## Past-directed scalar field gradients

We consider scalar-tensor theories described by the action [35]

1 $$\begin{aligned} S_\textrm{ST} = \frac{1}{16\pi } \int d^4x \sqrt{-g} \left[ \phi R -\frac{\omega (\phi )}{\phi } \, \nabla ^c\phi \nabla _c\phi -V(\phi ) \right] +S^\mathrm{(m)}, \end{aligned}$$

where *R* is the Ricci scalar, $V(\phi )$ the scalar field potential and $S^\text {(m)}=\int d^4x \sqrt{-g} \, \mathcal{L}^\text {(m)}$ the matter action. Let us define the timelike vector field $u^a$ as

2 $$\begin{aligned} u^a:= \frac{\nabla ^a \phi }{\sqrt{2 X}} , \qquad X:= - \frac{1}{2} \, \nabla _a \phi \nabla ^a \phi >0. \end{aligned}$$

Assuming that the spacetime manifold $(\mathcal {M}, g_{ab})$ admits a chart $(t, {\varvec{x}})$ with time coordinate *t*, then $g_{ab} \, u^a \, (\partial _t)^b > 0$ implies that $u^a$ is past-directed and it cannot be identified with the 4-velocity of an effective fluid, which is defined as timelike *future-directed* vector field.

Now, let the scalar field $\phi$ be such that $\nabla ^a \phi$ is past-directed: we can then define a future-directed vector field as

3 $$\begin{aligned} v^a:= - u^a = - \frac{\nabla ^a \phi }{\sqrt{2 X}} . \end{aligned}$$

The corresponding projection operator onto the 3-space orthogonal to $v^a$ is ${\mathfrak {h}^a}_b$, where

4 $$\begin{aligned} \mathfrak {h}_{ab}:= g_{ab} + v_a v_b = g_{ab} + u_a u_b = g_{ab} + \frac{\nabla _a \phi \nabla _b \phi }{2 X} =h_{ab} , \end{aligned}$$

and ${h^a}_b$ is the projection operator onto the 3-space orthogonal to $u^a$. Thus, $h_{ab}$ remains unaffected by the change of sign in the definition of the 4-velocity when the timelike gradient $\nabla ^a \phi$ is past-directed instead of being future-directed.

### Kinematic quantities

Let us examine now how the kinematic quantities associated with the effective scalar-tensor dissipative fluid [21–23] change when the definition of 4-velocity is modified to account for a past-directed gradient $\nabla ^a\phi$. In particular, we make explicit the relations between the kinematic quantities associated with $v^a$ (denoting them with $^{(v)}$) and those corresponding to $u^a=-v^a$ (denoting them with $^{(u)}$). For the 4-velocity gradient, we have

5 $$\begin{aligned} \nabla _a v_b = - \nabla _a u_b = - \frac{1}{\sqrt{2 X}} \left( \nabla _a \nabla _b \phi - \frac{\nabla _a X \nabla _b \phi }{2 X} \right) , \end{aligned}$$

which implies

6 $$\begin{aligned} \Theta _{(v)} = \nabla _a v^a = - \nabla _a u^a = - \Theta _{(u)} \end{aligned}$$

for the expansion scalar of the effective fluid,

7 $$\begin{aligned} a_{(v)} ^a:= v^c \nabla _c v^a = u^c \nabla _c u^a = a_{(u)} ^a \end{aligned}$$

for its 4-acceleration, while the projection of the velocity gradient onto the 3-space of the comoving observers reads

8 $$\begin{aligned} V^{(v)}_{ab}:= {h_a}^c {h_b}^d \nabla _d v_c = - {h_a}^c {h_b}^d \nabla _d u_c = - V^{(u)}_{ab} , \end{aligned}$$

and the new shear tensor is

9 $$\begin{aligned} \sigma ^{(v)} _{ab}:= V ^{(v)}_{(ab)} - \frac{\Theta ^{(v)} }{3} \, h_{ab} = - \left( V^{(u)} _{ab} - \frac{\Theta ^{(u)} }{3} \, h_{ab} \right) = - \sigma ^{(u)} _{ab} . \end{aligned}$$

These kinematic quantities do not depend on the field equations and are the same in all scalar-tensor gravity theories.

### Effective fluid stress-energy tensor

The effective energy-momentum tensor for scalar-tensor gravity reads

10 $$\begin{aligned} 8\pi T_{ab} = \frac{\omega }{\phi ^2} \left( \nabla _a \phi \nabla _b \phi - \frac{1}{2} \, g_{ab} \nabla ^c \phi \nabla _c \phi \right) + \frac{1}{\phi } \left( \nabla _a \nabla _b \phi -g_{ab} \square \phi \right) - \frac{V}{2 \phi } \, g_{ab}\nonumber \\ \end{aligned}$$

and it has been recognised to have the form of an imperfect fluid stress-energy tensor. In the case of past-directed gradients of $\phi$, we can write it as

11 $$\begin{aligned} T^{(v)}_{ab} = \rho ^{(v)} \, v_a v_b + q^{(v)}_a v_b + q^{(v)}_b v_a + \Pi ^{(v)}_{ab}, \end{aligned}$$

where the effective energy density, heat flux density, stress tensor, isotropic pressure, and anisotropic stress tensor (the trace-free part $\pi _{ab}$ of the stress tensor $\Pi _{ab}$) in the comoving frame of the effective fluid are, respectively,

$$\begin{aligned} \rho ^{(v)}= & {} T_{ab} v^a v^b, \\ q^{(v)} _a= & {} -T_{cd} \, v^c {h_a}^d, \\ \Pi ^{(v)}_{ab}= & {} P^{(v)} h_{ab} + \pi ^{(v)}_{ab} = T_{cd} \, {h_a}^c \, {h_b}^d, \\ P^{(v)}= & {} \frac{1}{3}\, g^{ab}\Pi ^{(v)}_{ab} =\frac{1}{3} \, h^{ab} T_{ab}, \\ \pi ^{(v)}_{ab}= & {} \Pi ^{(v)} _{ab} - P^{(v)}h_{ab}. \end{aligned}$$

It is straightforward to see that some of these quantities are not altered with respect to those arising from future-directed scalar field gradients:

12 $$\begin{aligned} \rho ^{(v)} = \rho ^{(u)}, \quad \quad \quad \Pi ^{(v)}_{ab} = \Pi ^{(u)}_{ab}, \quad \quad \quad P^{(v)} = P^{(u)}, \quad \quad \quad \pi ^{(v)}_{ab} = \pi ^{(u)}_{ab}. \end{aligned}$$

However, the heat flux density changes sign when the 4-velocity changes orientation:

13 $$\begin{aligned} q^{(v)} _a = -T_{cd} \, v^c {h_a}^d = T_{cd} \, u^c {h_a}^d = - q^{(u)} _a , \end{aligned}$$

which has important consequences for the definition of a meaningful temperature, as we detail in the following.

### First-order thermodynamics with past-directed scalar field gradients

Eckart’s thermodynamics provides a simple non-equilibrium thermodynamics that is first-order in the dissipative variables. Based on its three constitutive equations [25], we can find relationships between the kinematic quantities of the effective fluid and the dissipative thermodynamical variables, therefore building an analogy between the imperfect fluid and scalar-tensor gravity in a thermodynamical setting. The following relationships between the heat flux and the 4-acceleration, and the anisotropic stress and shear tensor, respectively, hold when dealing with future-directed gradients [24, 26, 27, 30]:

14 $$\begin{aligned} q_a^{(u)} = -\frac{ \sqrt{2X}}{ 8 \pi \phi } \, a^{(u)}_a, \quad \quad \quad \pi ^{(u)}_{ab} = \frac{ \sqrt{2X}}{ 8 \pi \phi } \, \sigma ^{(u)} _{ab}. \end{aligned}$$

Hence, given (12) and (13), we have

15 $$\begin{aligned} q^{(v)} _a = - q^{(u)} _a = \frac{ \sqrt{2X}}{ 8 \pi \phi } \, a^{(u)}_a = \frac{ \sqrt{2X}}{ 8 \pi \phi } \, a^{(v)}_a \end{aligned}$$

and

16 $$\begin{aligned} \pi ^{(v)}_{ab} = \pi ^{(u)}_{ab} = \frac{ \sqrt{2X}}{ 8 \pi \phi } \, \sigma ^{(u)} _{ab} = - \frac{ \sqrt{2X}}{ 8 \pi \phi } \, \sigma ^{(v)} _{ab} . \end{aligned}$$

This means that, *for a scalar field with timelike past-directed gradient*, one finds the “temperature of scalar-tensor gravity”

17 $$\begin{aligned} (\mathcal {K} \mathcal {T}) ^{(v)} = - (\mathcal {K} \mathcal {T}) ^{(u)} = - \frac{ \sqrt{2X}}{ 8 \pi \phi } < 0 \end{aligned}$$

(where $\mathcal{K}$ is the thermal conductivity, $\mathcal{T}$ is the temperature, and these two quantities always appear together in our analysis). Moreover, the shear viscosity coefficient $\eta$ defined by the constitutive relation $\pi _{ab} = -2\eta \, \sigma _{ab}$ [25] reads

18 $$\begin{aligned} \eta ^{(v)} = - \eta ^{(u)} = \frac{ \sqrt{2X}}{ 16 \pi \phi } > 0 . \end{aligned}$$

Thus, for past-directed gradients, we find a negative temperature and positive shear viscosity, opposite to the result for future-directed gradients. This is precisely the reason why making sure the velocity of $\phi$ is future-directed is crucial: the thermodynamical analogy built in [24, 26, 27] itself relies on a meaningful notion of temperature. The fact that such a temperature naturally arose to be positive-definite in the case of future-directed velocity is one of the promising features of the formalism. Moreover, modified gravity theories with degrees of freedom additional to those of GR always have a positive temperature with respect to GR, which is quite intuitive. The only meaningful situation where we found a negative temperature was that of Nordström gravity, that possesses *less* degrees of freedom than GR [32]. Additionally, the negative shear viscosity previously found in [24, 27] made sense as there is no reason to expect the effective fluid we are dealing with to be isolated. In the present work, a positive $\eta$ corresponds to the more usual case of an isolated fluid.

The previous findings [24, 26, 27, 30] remain valid, provided that one restricts the application of our formalism to scalar fields with future-directed timelike gradients.

### Approach to thermal equilibrium

Let us compute now the evolution of $(\mathcal {K} \mathcal {T})^{(v)}$ with respect to the time direction dictated by $v^a$, which is given by [24, 26, 27]

19 $$\begin{aligned} \begin{aligned} \frac{d}{d\tau } (\mathcal {K} \mathcal {T}) ^{(v)}&= v^a \nabla _a (\mathcal {K} \mathcal {T}) ^{(v)} = (- u^a) \nabla _a \left[ - (\mathcal {K} \mathcal {T}) ^{(u)} \right] = u^a \nabla _a (\mathcal {K} \mathcal {T}) ^{(u)} \\&= 8\pi (\mathcal {K} \mathcal {T}) _{(u)} ^2 - \Theta _{(u)} (\mathcal {K} \mathcal {T}) _{(u)} + \frac{\Box \phi }{8 \pi \phi } \\&= 8\pi (\mathcal {K} \mathcal {T}) _{(v)} ^2 - \Theta _{(v)} (\mathcal {K} \mathcal {T}) _{(v)} + \frac{\Box \phi }{8 \pi \phi } . \end{aligned} \end{aligned}$$

That is, the effective heat equation describing the approach to (or the departure from) thermal equilibrium in the first-order thermodynamics of scalar-tensor gravity reads

20 $$\begin{aligned} \frac{d}{d\tau } (\mathcal {K} \mathcal {T}) ^{(v)} = 8\pi (\mathcal {K} \mathcal {T}) _{(v)} ^2 - \Theta _{(v)} (\mathcal {K} \mathcal {T}) _{(v)} + \frac{\Box \phi }{8 \pi \phi } \end{aligned}$$

and is, therefore, not affected by the replacement $u^a \mapsto -u^a =v^a$.

## Revisiting the Brans-Dicke dust solution

Many analytical solutions of scalar-tensor gravity are known in various physical contexts, ranging from FLRW cosmology (*e.g.*, [35]) to spherically symmetric solutions describing black holes and other objects (see [36] for a recent review). A large fraction of the literature on Horndeski and DHOST gravity is devoted to the search of such solutions with disformal (and other) techniques [37–51]. These solutions were of course derived regardless of the effective fluid formalism and the first-order thermodynamics approach which, *per se*, do not offer new methods for solving the field equations exactly (although they do offer novel physical interpretations [52]).

Therefore, certain scalar-tensor solutions feature timelike and past-directed gradient $\nabla ^a \phi$ of the scalar degree of freedom. Systematically searching for these solutions and listing them would not be particularly illuminating; rather, we focus on a classic simple FLRW solution that has been known for a long time, namely the Brans-Dicke dust solution [6], which we analysed in [30]. This is the only solution we studied through the lens of first-order thermodynamics whose study requires to be revisited in light of the extension to past-directed fluid velocity that we provide in this paper.

This solution describes a matter-dominated universe permeated by a pressureless dust fluid in Brans-Dicke gravity [6] with $V(\phi )=0$ and $\omega \ne -4/3$ and reads

21 $$\begin{aligned} a(t)=a_0 \, t^q, \quad \quad \quad \phi (t)=\phi _0 \, t^s, \quad \quad \quad \rho ^\mathrm {(m)}(t)= \rho _0 \, t^r, \end{aligned}$$

where *a*(*t*) is the cosmic scale factor, $\rho ^{(\mathrm m)}=\rho ^{(\mathrm m)}(t)$ the matter energy density, $\rho _0= C/a_0^3$, *C* is an integration constant related to the initial conditions, and

22 $$\begin{aligned} q=\dfrac{2(\omega +1)}{3\omega +4}, \quad \quad \quad s=\dfrac{2}{3\omega +4}, \quad \quad \quad r=-3q \end{aligned}$$

satisfy $3q+s=2$. Then, if the dot denotes differentiation with respect to *t*, we find

23 $$\begin{aligned} \dot{\phi } = \frac{s}{t} \, \phi . \end{aligned}$$

Since $\phi >0$, in order to have $\dot{\phi }>0$ one has to require $s>0$, which implies $\omega >-4/3$. From these assumptions, it follows that

24 $$\begin{aligned} \nabla ^a \phi = g^{a0} \dot{\phi } = g^{a0} \, \frac{s \phi }{t} , \end{aligned}$$

which implies^2^$\nabla ^0 \phi = - s\phi /t <0$. Therefore $\nabla ^a \phi$ is past-directed.

The 4-velocity of the effective fluid must therefore be defined as

25 $$\begin{aligned} v^a = - u^a = - \frac{\nabla ^a \phi }{\sqrt{2X}} , \quad 2 X:= - \nabla ^a \phi \nabla _a \phi = \frac{s^2 \phi ^2}{t^2} . \end{aligned}$$

The product of the temperature and the thermal conductivity is therefore negative and diverges as the initial cosmological singularity is approached

26 $$\begin{aligned} (\mathcal {K} \mathcal {T}) ^{(v)} = - \frac{ \sqrt{2X}}{ 8 \pi \phi } = - \frac{s}{8 \pi \, t} \underset{t \rightarrow 0^+}{\longrightarrow }\ - \infty . \end{aligned}$$

The shear viscosity $\eta ^{(v)}$ vanishes because of the symmetries of FLRW (the heat flux $q^{(v)}$ would vanish too, but in [30] we chose the heat flux as a timelike vector aligned with the four-velocity of comoving observers).

## Conclusions

In this work we extend the first-order thermodynamics of scalar-tensor gravity to timelike and past-directed gradients of the scalar field. The first-order thermodynamics is a recent approach that aims to provide a novel understanding of the intriguing relationship between thermodynamics and gravity, by characterising GR as a zero-temperature equilibrium state and any modified gravity with additional degrees of freedom than GR as a non-equilibrium state. This idea depicts a “thermodynamics of gravitational theories" and is based on the imperfect fluid description of scalar-tensor theories.

Since the whole picture relies on the analogy of modified theories with an effective scalar field fluid that is supposed to have a meaningful (i.e. future-directed) 4-velocity, previous works had not considered the possibility of past-directed velocity.

Past-directed scalar field gradients do arise in analytical solutions of scalar-tensor gravity and it is not surprising that the corresponding temperatures become negative, which seems natural from the thermodynamic point of view. Time reversal $t \rightarrow -t$ achieves the same result, turning future-directed scalar field gradients into past-directed ones. This feature corresponds to the deterministic character of “standard” gravity, to go beyond which one would have to concoct a theory of gravity originating from stochastic dynamics: then time-reversal would not simply turn future-directed into past-directed $\nabla ^a\phi$. Unfortunately, stochastic semiclassical gravity and the theoretical efforts on the Einstein-Langevin equation (see [53] for a review) are quite remote from the first-order thermodynamics of classical gravity, with no obvious connection.

The present work addresses past-directed scalar field gradients: we find that the kinematic fluid quantities remain unchanged, but certain thermodynamical variables such as heat fluxes change sign, leading to a negative temperature and a positive shear viscosity, at variance with previous works. These results are also confirmed by revisiting the Brans-Dicke dust solution, which does have a past-directed fluid velocity.

A negative temperature is problematic in first-order thermodynamics, where additional degrees of freedom to those of GR give modified theories a positive-definite temperature. We cannot provide an assessment of the physical viability of solutions in scalar-tensor gravity through the sign of the temperature within our formalism, but we are now aware of the need to restrict upcoming analyses to situations with future-directed scalar field velocity only, if the thermodynamical formalism is to meaningfully hold.

## Data Availability

Not applicable.

## Footnotes

1 The idea of modified (specifically, quadratic *f*(*R*)) gravity as a state of non-equilibrium, with GR as the equilibrium state, goes back to Jacobson’s thermodynamics of spacetime [28, 29]. However, in spite of many studies, a “temperature of gravity” was never identified in this context, which is entirely different from the first-order thermodynamics of scalar-tensor gravity discussed here.

2 $s>0$ implies an increasing $\phi$ and an effective gravitational coupling $G_\textrm{eff} \simeq \phi ^{-1}$ decreasing with time. *A priori* one would expect the weakening of gravity to characterize stability but the past-pointing $\nabla ^a \phi$ seems to defy this intuition.

## References

[1] Callan CG, Martinec EJ, Perry MJ, Friedan D (1985). Strings in background fields. Nucl. Phys. B.

[2] Fradkin, E.S., Tseytlin, A.A.: Quantum string theory effective action. Nucl. Phys. B **261**, 1–27 (1985). 10.1016/0550-3213(85)90559-0. [Erratum: Nucl. Phys. B 269, 745-745 (1986)]

[3] Amendola L, Tsujikawa S (2010). Dark energy theory and observations.

[4] Riess, A.G.: The Expansion of the Universe is Faster than Expected. Nature Rev. Phys. **2**(1), 10–12 (2019) arXiv:2001.03624 [astro-ph.CO]. 10.1038/s42254-019-0137-0

[5] Di Valentino, E., Mena, O., Pan, S., Visinelli, L., Yang, W., Melchiorri, A., Mota, D.F., Riess, A.G., Silk, J.: In the realm of the Hubble tension-a review of solutions. Class. Quant. Grav. **38**(15), 153001 (2021) arXiv:2103.01183 [astro-ph.CO]. 10.1088/1361-6382/ac086d

[6] Brans C, Dicke RH (1961). Mach’s principle and a relativistic theory of gravitation. Phys. Rev..

[7] Bergmann PG (1968). Comments on the scalar tensor theory. Int. J. Theor. Phys..

[8] Nordtvedt K (1968). Equivalence Principle for Massive Bodies 2. Theory. Phys. Rev..

[9] Wagoner RV (1970). Scalar tensor theory and gravitational waves. Phys. Rev. D.

[10] Nordtvedt K (1970). PostNewtonian metric for a general class of scalar tensor gravitational theories and observational consequences. Astrophys. J..

[11] Capozziello S, Carloni S, Troisi A (2003). Quintessence without scalar fields. Recent Res. Dev. Astron. Astrophys..

[12] Sotiriou, T.P., Faraoni, V.: f(R) Theories Of Gravity. Rev. Mod. Phys. **82**, 451–497 (2010) arXiv:0805.1726 [gr-qc]. 10.1103/RevModPhys.82.451

[13] De Felice, A., Tsujikawa, S.: f(R) theories. Living Rev. Rel. **13**, 3 (2010) arXiv:1002.4928 [gr-qc]. 10.12942/lrr-2010-3 10.12942/lrr-2010-3 PMC5255939 28179828

[14] Nojiri, S., Odintsov, S.D.: Unified cosmic history in modified gravity: from F(R) theory to Lorentz non-invariant models. Phys. Rept. **505**, 59–144 (2011) arXiv:1011.0544 [gr-qc]. 10.1016/j.physrep.2011.04.001

[15] Horndeski GW (1974). Second-order scalar-tensor field equations in a four-dimensional space. Int. J. Theor. Phys..

[16] Kobayashi, T.: Horndeski theory and beyond: a review. Rept. Prog. Phys. **82**(8), 086901 (2019) arXiv:1901.07183 [gr-qc]. 10.1088/1361-6633/ab2429 10.1088/1361-6633/ab2429 31121569

[17] Langlois, D.: Dark energy and modified gravity in degenerate higher-order scalar-tensor (DHOST) theories: a review. Int. J. Mod. Phys. D **28**(05), 1942006 (2019) arXiv:1811.06271 [gr-qc]. 10.1142/S0218271819420069

[18] Abbott, B.P., et al.: GW170817: Observation of gravitational waves from a Binary neutron star inspiral. Phys. Rev. Lett. **119**(16), 161101 (2017) arXiv:1710.05832 [gr-qc]. 10.1103/PhysRevLett.119.161101 10.1103/PhysRevLett.119.161101 29099225

[19] Abbott, B.P., et al.: Gravitational waves and gamma-rays from a binary neutron star merger: GW170817 and GRB 170817A. Astrophys. J. Lett. **848**(2), 13 (2017) arXiv:1710.05834 [astro-ph.HE]. 10.3847/2041-8213/aa920c

[20] Langlois, D., Saito, R., Yamauchi, D., Noui, K.: Scalar-tensor theories and modified gravity in the wake of GW170817. Phys. Rev. D **97**(6), 061501 (2018) arXiv:1711.07403 [gr-qc]. 10.1103/PhysRevD.97.061501

[21] Pimentel LO (1989). Energy momentum tensor in the general Scalar–Tensor theory. Class. Quant. Grav..

[22] Faraoni, V., Coté, J.: Imperfect fluid description of modified gravities. Phys. Rev. D **98**(8), 084019 (2018) arXiv:1808.02427 [gr-qc]. 10.1103/PhysRevD.98.084019

[23] Nucamendi, U., De Arcia, R., Gonzalez, T., Horta-Rangel, F.A., Quiros, I.: Equivalence between Horndeski and beyond Horndeski theories and imperfect fluids. Phys. Rev. D **102**(8), 084054 (2020) arXiv:1910.13026 [gr-qc]. 10.1103/PhysRevD.102.084054

[24] Giusti, A., Zentarra, S., Heisenberg, L., Faraoni, V.: First-order thermodynamics of Horndeski gravity. Phys. Rev. D **105**(12), 124011 (2022) arXiv:2108.10706 [gr-qc]. 10.1103/PhysRevD.105.124011

[25] Eckart C (1940). The thermodynamics of irreversible processes 3. Relativistic theory of the simple fluid. Phys. Rev..

[26] Faraoni, V., Giusti, A.: Thermodynamics of scalar-tensor gravity. Phys. Rev. D **103**(12), 121501 (2021) arXiv:2103.05389 [gr-qc]. 10.1103/PhysRevD.103.L121501

[27] Faraoni, V., Giusti, A., Mentrelli, A.: New approach to the thermodynamics of scalar-tensor gravity. Phys. Rev. D **104**(12), 124031 (2021) arXiv:2110.02368 [gr-qc]. 10.1103/PhysRevD.104.124031

[28] Jacobson, T.: Thermodynamics of space-time: The Einstein equation of state. Phys. Rev. Lett. **75**, 1260–1263 (1995) arXiv:gr-qc/9504004. 10.1103/PhysRevLett.75.1260 10.1103/PhysRevLett.75.1260 10060248

[29] Eling, C., Guedens, R., Jacobson, T.: Non-equilibrium thermodynamics of spacetime. Phys. Rev. Lett. **96**, 121301 (2006) arXiv:gr-qc/0602001. 10.1103/PhysRevLett.96.121301 10.1103/PhysRevLett.96.121301 16605892

[30] Giardino, S., Faraoni, V., Giusti, A.: First-order thermodynamics of scalar-tensor cosmology. JCAP **04**(04), 053 (2022) arXiv:2202.07393 [gr-qc]. 10.1088/1475-7516/2022/04/053

[31] Faraoni, V., Françonnet, T.B.: Stealth metastable state of scalar-tensor thermodynamics. Phys. Rev. D **105**(10), 104006 (2022) arXiv:2203.14934 [gr-qc]. 10.1103/PhysRevD.105.104006

[32] Faraoni, V., Giusti, A., Jose, S., Giardino, S.: Peculiar thermal states in the first-order thermodynamics of gravity. Phys. Rev. D **106**(2), 024049 (2022) arXiv:2206.02046 [gr-qc]. 10.1103/PhysRevD.106.024049

[33] Faraoni, V., Giardino, S., Giusti, A., Vanderwee, R.: Scalar field as a perfect fluid: thermodynamics of minimally coupled scalars and Einstein frame scalar-tensor gravity (2022) arXiv:2208.04051 [gr-qc] 10.1140/epjc/s10052-023-11186-7 PMC9842576 36683948

[34] Wald, R.M.: General Relativity. Chicago Univ. Press, Chicago, USA (1984). 10.7208/chicago/9780226870373.001.0001

[35] Faraoni, V.: Cosmology in Scalar-Tensor Gravity. Kluwer Academic, Dordrecht, The Netherlands (2004). 10.1007/978-1-4020-1989-0

[36] Faraoni, V., Giusti, A., Fahim, B.H.: Spherical inhomogeneous solutions of Einstein and scalar–tensor gravity: A map of the land. Phys. Rept. **925**, 1–58 (2021) arXiv:2101.00266 [gr-qc]. 10.1016/j.physrep.2021.04.003

[37] Ben Achour, J., Liu, H., Mukohyama, S.: Hairy black holes in DHOST theories: Exploring disformal transformation as a solution-generating method. JCAP **02**, 023 (2020) arXiv:1910.11017 [gr-qc]. 10.1088/1475-7516/2020/02/023

[38] Faraoni, V., Leblanc, A.: Disformal mappings of spherical DHOST geometries. JCAP **08**, 037 (2021) arXiv:2107.03456 [gr-qc]. 10.1088/1475-7516/2021/08/037

[39] Achour, J.B., De Felice, A., Gorji, M.A., Mukohyama, S., Pookkillath, M.C.: Disformal map and Petrov classification in modified gravity. JCAP **10**, 067 (2021) arXiv:2107.02386 [gr-qc]. 10.1088/1475-7516/2021/10/067

[40] Babichev, E., Esposito-Farèse, G.: Time-Dependent Spherically Symmetric Covariant Galileons. Phys. Rev. D **87**, 044032 (2013) arXiv:1212.1394 [gr-qc]. 10.1103/PhysRevD.87.044032

[41] Anabalon, A., Cisterna, A., Oliva, J.: Asymptotically locally AdS and flat black holes in Horndeski theory. Phys. Rev. D **89**, 084050 (2014) arXiv:1312.3597 [gr-qc]. 10.1103/PhysRevD.89.084050

[42] Babichev, E., Charmousis, C.: Dressing a black hole with a time-dependent Galileon. JHEP **08**, 106 (2014) arXiv:1312.3204 [gr-qc]. 10.1007/JHEP08(2014)106

[43] Charmousis, C., Kolyvaris, T., Papantonopoulos, E., Tsoukalas, M.: Black Holes in Bi-scalar Extensions of Horndeski Theories. JHEP **07**, 085 (2014) arXiv:1404.1024 [gr-qc]. 10.1007/JHEP07(2014)085

[44] Kobayashi, T., Tanahashi, N.: Exact black hole solutions in shift symmetric scalar-tensor theories. PTEP **2014**, 073–02 (2014) arXiv:1403.4364 [gr-qc]. 10.1093/ptep/ptu096

[45] Babichev, E., Esposito-Farese, G.: Cosmological self-tuning and local solutions in generalized Horndeski theories. Phys. Rev. D **95**(2), 024020 (2017) arXiv:1609.09798 [gr-qc]. 10.1103/PhysRevD.95.024020

[46] Motohashi, H., Minamitsuji, M.: General Relativity solutions in modified gravity. Phys. Lett. B **781**, 728–734 (2018) arXiv:1804.01731 [gr-qc]. 10.1016/j.physletb.2018.04.041

[47] Babichev, E., Charmousis, C., Lehébel, A.: Asymptotically flat black holes in Horndeski theory and beyond. JCAP **04**, 027 (2017) arXiv:1702.01938 [gr-qc]. 10.1088/1475-7516/2017/04/027

[48] Anson T, Babichev E, Charmousis C, Hassaine M (2021). Disforming the Kerr metric.. JHEP.

[49] Ben Achour, J., Liu, H., Motohashi, H., Mukohyama, S., Noui, K.: On rotating black holes in DHOST theories. JCAP **11**, 001 (2020) arXiv:2006.07245 [gr-qc]. 10.1088/1475-7516/2020/11/001

[50] Chatzifotis, N., Papantonopoulos, E., Vlachos, C.: Disformal transition of a black hole to a wormhole in scalar-tensor Horndeski theory. Phys. Rev. D **105**(6), 064025 (2022) arXiv:2111.08773 [gr-qc]. 10.1103/PhysRevD.105.064025

[51] Saadati, R., Giusti, A., Faraoni, V., Shojai, F.: New time-dependent solutions of viable Horndeski gravity. JCAP **09**, 067 (2022) arXiv:2207.07060 [gr-qc]. 10.1088/1475-7516/2022/09/067

[52] Miranda, M., Vernieri, D., Capozziello, S., Faraoni, V.: Fluid nature constrains Horndeski gravity (2022) arXiv:2209.02727 [gr-qc]

[53] Hu, B.L., Verdaguer, E.: Stochastic Gravity: Theory and Applications. Living Rev. Rel. **11**, 3 (2008) arXiv:0802.0658 [gr-qc]. 10.12942/lrr-2008-3 10.12942/lrr-2004-3 PMC5660882 29142503

